# Tungsten Trioxide (WO_3_)-assisted Photocatalytic Degradation of Amoxicillin by Simulated Solar Irradiation

**DOI:** 10.1038/s41598-019-45644-8

**Published:** 2019-06-27

**Authors:** Thao Thi Nguyen, Seong-Nam Nam, Jooyoung Son, Jeill Oh

**Affiliations:** Department of Civil and Environmental Engineering, Chung-Ang University, 84, Heukseok-ro, Dongjak-gu, Seoul 06974 Republic of Korea

**Keywords:** Hydrology, Pollution remediation

## Abstract

This study investigates the photocatalytic degradation of amoxicillin (AMO) by simulated solar irradiation using WO_3_ as a catalyst. A three-factor-three-level Box-Behnken design (BBD) consisting of 30 experimental runs is employed with three independent variables: initial AMO concentration, catalyst dosage, and pH. The experimental results are analyzed in terms of AMO degradation and mineralization, the latter of which is measured using dissolved organic carbon (DOC). The results show that the photocatalytic degradation of AMO follows pseudo-first-order kinetics. AMO degradation efficiency and the pseudo-first-order rate constants decrease with increasing initial AMO concentration and pH and increase with increasing catalyst dosage. Though AMO degradation is almost fully complete under the experimental conditions, DOC removal is much lower; the highest DOC removal rate is 35.82% after 180 min. Using these experimental results, second-order polynomial response surface models for AMO and DOC removal are constructed. In the AMO removal model, the first-order terms are the most significant contributors to the prediction, followed by the quadratic and interaction terms. Initial AMO concentration and pH have a significant negative impact on the photocatalytic degradation of AMO, while catalyst dosage has a significant positive impact. In contrast, in the DOC removal model, the quadratic terms make the most significant contribution to the prediction and the first-order terms the least. The optimal conditions for the photocatalytic degradation of AMO are found to be an initial AMO concentration of 1.0 μM, a catalyst dosage of 0.104 g/L, and a pH of 4, under which almost complete removal of AMO is achieved (99.99%).

## Introduction

Amoxicillin (AMO), a moderate-spectrum bacteriolytic β-lactam antibiotic, was one of the most frequently used antimicrobial agents in 71 countries between 2000 and 2010^1^. It is also the most commonly prescribed antibiotic in South Korea, with approximately 97 tons of AMO produced annually^2^. AMO is on the World Health Organization’s List of Essential Medicines, a list of the most important medications needed in a basic health system^3^. After ingestion, it is excreted in urine and feces and enters aqueous environments through the water cycle (Fig. S1**)**^4^. AMO has been detected in various sources, including wastewater treatment plants (30–6,940 ng/L) and raw sewage (280 ng/L) in Australia^5,6^; wastewater treatment plants (1.80–622 ng/L) in Italy^7–9^; urban effluent (1,670 ng/L) in Spain^10^; surface water in Australia (200 ng/L)^6^, the UK (39–245 ng/L)^11^, and South Korea (0.02–1.07 µg/L)^12^; and hospital effluent in Australia (900 ng/L)^6^, Spain^10^, and Germany (28.0–82.7 µg/L)^13^. The presence of high levels of AMO has potential risks for ecosystems and human health. For example, toxic effects of AMO on aquatic organisms have been reported in a number of studies, including the blue-green alga *Synechococcus leopoliensis*^7^ and the cyanobacterium *Microcystis aeruginosa*^14^. AMO has also been reported to cause the premature hatching, malformation, and changes in enzyme activity in both embryonic and adult zebrafish (*Danio rerio*)^15^ and to induce oxidative stress in the brain, gills, liver, and kidneys of common carp (*Cyprinus carpio*)^16^.

With the increased use of AMO worldwide, AMO levels in water and wastewater are expected to rise, so the effective removal of AMO using wastewater treatment processes has become more important. However, conventional treatment processes, which mostly rely on biological treatment, are not effective in eliminating AMO. Therefore, it is necessary to develop new alternatives to remove this pollutant from wastewater. In recent years, various wastewater treatment methods have been proposed for AMO degradation; a subset of these, advanced oxidation processes (AOPs), has been found to be highly efficient in removing AMO from wastewater. AOPs rely on the *in-situ* production of highly reactive hydroxyl radicals (^•^OH) with the help of one or more primary oxidants (e.g., ozone, hydrogen peroxide, and oxygen), energy sources (e.g. ultraviolet, solar and visible light), and/or catalysts (e.g. tungsten trioxide, and titanium dioxide)^17–19^. Refractory pollutants in water react with ^•^OH, leading to their decomposition or mineralization into CO_2_, H_2_O, and inorganic ions^17–19^. Previous studies of AOPs, some of which are summarized in Table 1, have introduced methods such as ozonation^20^, Fenton^21^ and photo-Fenton^22^, UV or solar photolysis with catalysts such as TiO_2_ and ZnO^23–27^). In particular, heterogeneous photocatalysis has shown considerable potential as a versatile, low-cost, environmentally friendly, and sustainable AOP treatment technology for the removal of emerging contaminants, including AMO.

**Table 1:** Amoxicillin (AMO) degradation by advanced oxidation processes (AOPs).

AOP types	Experimental conditions	Reaction time (min)	AMO removal (%)	Mineralization (%)	References
Ozonation	[AMO] = 5.0 × 10^−4^ M, O_2_ flowrate = 36 L/h, [O_3_] = 1.6 × 10^−4^ M, pH = 2.5–7.2	20	>90	18.2	^20^
Fenton	[AMO] = 105 mg/L, [Fe^2+^] = 25 mg/L, [H_2_O_2_] = 255 mg/L	15	100	37	^21^
Photo-Fenton	[AMO] = 104 mg/L, [H_2_O_2_]/[Fe^2+^] = 20, pH = 3, UVA (365 nm) = 6 W	50	100	58.4	^22^
UV photolysis	[AMO] = 104 mg/L, UVA (365 nm) = 6 W, pH = 5	300	2.9	NA	^23^
UV/H_2_O_2_	[AMO] = 100 μM, A low pressure Hg arc-UV lamp UVC (254 nm), [H_2_O_2_] = 10 mM, pH = 7	20	>99	<22% after 20 min 50% after 80 min	^24^
UV/TiO_2_	[AMO] = 104 mg/L, TiO_2_ = 1.0 g/L, UVA (365 nm) = 6 W, pH = 5	300	54.8	<3%	^23^
UV/H_2_O_2_/TiO_2_	[AMO] = 104 mg/L, TiO_2_ = 1.0 g/L, [H_2_O_2_] = 100 mg/L, UVA (365 nm) = 6 W, pH = 5	300	100% after 20 min	13.9	^23^
UV/ZnO	[AMO] = 104 mg/L, ZnO = 0.5 g/L, UVA (365 nm) = 6 W, pH = 11	180	100	9.7	^25^
Solar-Photolysis	[AMO] = 17 mg/L, Pilot plant with compound parabolic collectors, pH = 9.5	240	14.32	NA	^26^
Solar/TiO_2_	[AMO] = 100 mg/L, TiO_2_ = 1.0 g/L, Natural sunlight, 16 mW/cm^2^, pH = 6	120	80	NA	^27^

Heterogeneous photocatalysis involves the irradiation of a semiconductor catalyst (e.g. TiO_2_, ZnO, WO_3_, Fe_2_O_3_, CdO, CdS, GaS, GaP, SnO_2_, ZnS, and SrTiO_3_) with a light source (i.e., ultraviolet, solar, and visible light) to generate highly reactive transitory species (i.e., ^•^OH, ${{\rm{O}}}_{2}^{\bullet -}$) for the subsequent mineralization of organic pollutants. According to^28–31^, photocatalytic degradation reactions are initiated when the semiconductor catalyst absorbs photons from visible light. Upon the absorption of light, the electrons in the valence band (VB) of the catalyst are transferred to the conduction band (CB), generating an electron-hole pair. This leads to the formation of ^•^OH via oxidation, with H_2_O or OH^−^ molecules reacting with VB holes $({{\rm{h}}}_{{\rm{VB}}}^{+})$, while superoxide radicals $({{\rm{O}}}_{2}^{\bullet -})$ from dissolved O_2_ and CB electrons $({{\rm{e}}}_{{\rm{CB}}}^{-})$ are formed via reduction. These active radicals are then able to break down organic contaminants in aqueous solutions. The majority of AMO photodegradation approaches use the direct excitation of molecules by UV light (e.g., UV/TiO_2_ and UV/ZnO)^23,25,30^. However, the use of UV light in the treatment of large volumes of industrial effluent is not feasible or economical. Hence, researchers have focused on using simulated sunlight and developing solar/visible photocatalysts, which exhibit high activity levels under solar and visible light radiation. Because solar energy is a cheap, abundant, non-polluting, renewable, and readily available energy source in most parts of the world^32^, solar photocatalysis has become a target green technology for the treatment of water and wastewater.

Though a few studies have focused on the degradation of AMO via solar photocatalysis using TiO_2_^26,27,33^, no research on visible/solar photocatalysis assisted by tungsten trioxide (WO_3_) has been reported to date. WO_3_, which has a narrow band gap energy (2.4–2.8 eV), is a visible light responsive catalyst with stable physicochemical properties^34–38^. WO_3_ is physiochemically stable and is mechanically robust in aqueous solutions, while the production of high-purity WO_3_ is relatively facile and cost-effective, making it a suitable choice for the photocatalytic degradation of organic pollutants under solar irradiation^34–41^. Therefore, the objective of this study is to employ WO_3_-assisted photocatalysis in a simulated solar irradiation system to facilitate AMO degradation.

**Figure 1:**
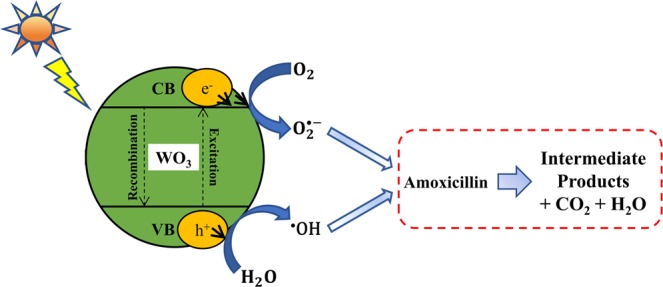
Proposed mechanisms for the solar photocatalytic degradation of AMO.

The proposed reaction mechanisms for the WO_3_-assisted photocatalytic degradation of AMO under simulated solar irradiation are presented in Fig. 1 as the set of equations Eqs (1.1) to Eq. (1.11) ^40–42^. The VB holes of WO_3_ have a high oxidation power (E_VB_ = + 3.1–3.2 V_NHE_), where E_VB_ is the valance band edge at the normal hydrogen electrode (NHE), which facilitates the oxidation of water $({{\rm{E}}}_{{{\rm{H}}}_{2}{\rm{O}}/{{\rm{O}}}_{2}}=+\,1.23\,{{\rm{V}}}_{{\rm{NHE}}})$ via photogenerated ${{\rm{h}}}_{{\rm{VB}}}^{+}$ entities and the formation of ^•^OH, as presented in Eqs (1.3) and (1.4) ^33,40,43–45^. The reduction of dissolved or adsorbed O_2_ to superoxide anions radicals $({{\rm{O}}}_{2}^{\bullet -})$ by CB electrons ${(e}_{{\rm{CB}}}^{-})$ is outlined in Eq. (1.5). ${{\rm{O}}}_{2}^{\bullet -}$ is converted to hydrogen peroxide (H_2_O_2_) via disproportionation with protons (Eq. 1.6) or it takes the form of ${{\rm{HO}}}_{2}^{\bullet }$ via protonation, whose lifetime is short due to the rapid reaction with ${{\rm{O}}}_{2}^{\bullet -}$ or ${{\rm{HO}}}_{2}^{\bullet }$ to form stable H_2_O_2_ (Eqs 1.7 and 1.8)^46^. One-electron reduction of H_2_O_2_ produces ^•^OH (Eq. 1.9). Thus, the formation of ^•^OH can occur in two ways: via the oxidation of H_2_O or OH^−^ molecules or the reduction of O_2_. The formation and existence of oxidizing species depend on various factors, such as the potential of the CB edge (E_CB_), the pH at the zero point charge (pH_ZPC_) for the photocatalyst, and the pH of the medium itself. In general, a suitable E_CB_ (i.e., reduction potential) for the ${{\rm{O}}}_{2}/{{\rm{O}}}_{2}^{\bullet -}$ couple is E_CB_ = −0.28 V_NHE_, and a neutral or basic pH (≥7.0) supports the formation of ${{\rm{O}}}_{2}^{\bullet -}$, whereas a lower pH facilitates the formation of ^•^OH and H_2_O_2_^46,47^. Because WO_3_ has a lower E_CB_ (+0.3–0.5 V_NHE_), it cannot provide sufficient potential for the reduction of O_2_^40–45^. Consequently, ^•^OH is primarily generated via oxidative paths, after which, due to its strong oxidizing ability, it breaks AMO down into smaller intermediates or inorganics such as CO_2_ and H_2_O.

1.1 $${{\rm{WO}}}_{3}+{\rm{hv}}\to {{\rm{h}}}_{{\rm{VB}}}^{+}+{{\rm{e}}}_{{\rm{CB}}}^{-}$$

1.2 $${{\rm{H}}}_{2}{\rm{O}}\to {{\rm{OH}}}^{-}+{{\rm{H}}}^{+}$$

1.3 $${{\rm{h}}}_{{\rm{VB}}}^{+}+{{\rm{H}}}_{2}{\rm{O}}\to {}^{\bullet }{\rm{OH}}({\rm{hydroxyl}}\,{\rm{radical}})+{{\rm{H}}}^{+}$$

1.4 $${{\rm{h}}}_{{\rm{VB}}}^{+}+{{\rm{OH}}}^{-}\to {}^{\bullet }{\rm{OH}}({\rm{hydroxyl}}\,{\rm{radical}})$$

1.5 $${{\rm{e}}}_{{\rm{CB}}}^{-}+{{\rm{O}}}_{2}\to {{\rm{O}}}_{2}^{\bullet -}({\rm{superoxide}}\,{\rm{radical}})$$

1.6 $${{\rm{O}}}_{2}^{\bullet -}+2{{\rm{H}}}^{+}+{{\rm{e}}}_{{\rm{CB}}}^{-}\to {{\rm{H}}}_{2}{{\rm{O}}}_{2}$$

1.7 $${{\rm{O}}}_{2}^{\bullet -}+{{\rm{H}}}^{+}\to {{\rm{HO}}}_{2}^{\bullet }$$

1.8 $${{\rm{HO}}}_{2}^{\bullet }+{{\rm{HO}}}_{2}^{\bullet }\to {{\rm{H}}}_{2}{{\rm{O}}}_{2}+\frac{1}{2}{{\rm{O}}}_{2}$$

1.9 $${{\rm{H}}}_{2}{{\rm{O}}}_{2}+{{\rm{H}}}^{+}+{{\rm{e}}}_{{\rm{CB}}}^{-}\to {}^{\bullet }{\rm{OH}}+{{\rm{H}}}_{2}{\rm{O}}$$

1.10 $${}^{\bullet }{\rm{OH}}+{\rm{Amoxicillin}}\to {\rm{Intermediates}}+{{\rm{H}}}_{2}{\rm{O}}+{{\rm{CO}}}_{2}$$

1.11 $${{\rm{O}}}_{2}^{\bullet -}+{\rm{Amoxicillin}}\to {\rm{Intermediates}}+{{\rm{H}}}_{2}{\rm{O}}+{{\rm{CO}}}_{2}$$

The efficiency of this process depends on several parameters, such as the initial concentration of AMO, the catalyst dosage, pH, and the presence of competing radical scavengers. In order to understand the effects of these parameters on the degradation of AMO and to optimize the experimental conditions, we apply response surface methodology (RSM), a technique commonly used in process analysis and modeling.

RSM is a collection of mathematical and statistical techniques that are useful for developing, improving, and optimizing various processes. It can be used to evaluate the relative significance of several factors even in the presence of complex interactions^48^. The main objective of RSM is to determine the optimal operating conditions for a given system or to determine a region that satisfies operating specifications. Hence, RSM allows the optimal conditions for various reactions to be identified to reduce time, labor, and material costs. RSM also quantifies the relationships between controllable input parameters and obtained response surfaces. An adequate number of experiments is required to develop a mathematical model for predicting degradation efficiency and to determine the direct and interactive effects of the operating conditions. Central composite design (CCD) and Box–Behnken design (BBD) are the two most common experimental design methods for RSM^49,50^. The BBD is more convenient and less expensive than the CCD for the same number of factors. Therefore, we utilize RSM-BBD to optimize the operating conditions and increase the efficiency of AMO degradation.

In summary, this study aims to (i) assess the AMO degradation and mineralization efficiency of simulated solar irradiation using WO_3_ as a catalyst, (ii) examine the effects of different operating conditions (initial AMO concentration, catalyst dosage, and initial pH) on the photocatalytic degradation of AMO, and (iii) optimize the operating conditions based on RSM-BBD.

## Experimental

### Chemicals and reagents

Amoxicillin (CAS No. 26787-78-0, MF: C_16_H_19_N_3_O_5_S, MW: 365.40 g/mol) and tungsten (VI) oxide (CAS No. 1314-35-8, MF: WO_3_, MW: 231.84 g/mol, powder ≤ 0.20 µm) were purchased from Sigma-Aldrich, US. The properties of AMO are provided in Table 2. Sodium hydroxide was obtained from Deajung Chemicals (South Korea). Sulfuric acid (purity ≥ 96%) was purchased from Kanto Chemicals (Japan). All other chemicals used in this study were of analytical grade. The stock solution of AMO and other solutions were prepared using de-ionized water (DI; ≥ 18.2 Ω·cm^−1^) and diluted as required.

**Table 2:** Physicochemical properties of AMO.

Parameter	Amoxicillin
Molecular formula	C_16_H_19_N_3_O_5_S
Molecular weight	365.40 g/mol
Chemical structure	
λ_max_	230 nm
Solubility	very soluble in water (water solubility = 3,430 mg/L), sparingly soluble in anhydrous ethanol, and very slightly soluble in acetone.
pK_a_	pK_a1_ = 2.7, pK_a2_ = 7.5, pK_a3_ = 9.6^34^

### Photocatalytic experiments

**Figure 2:**
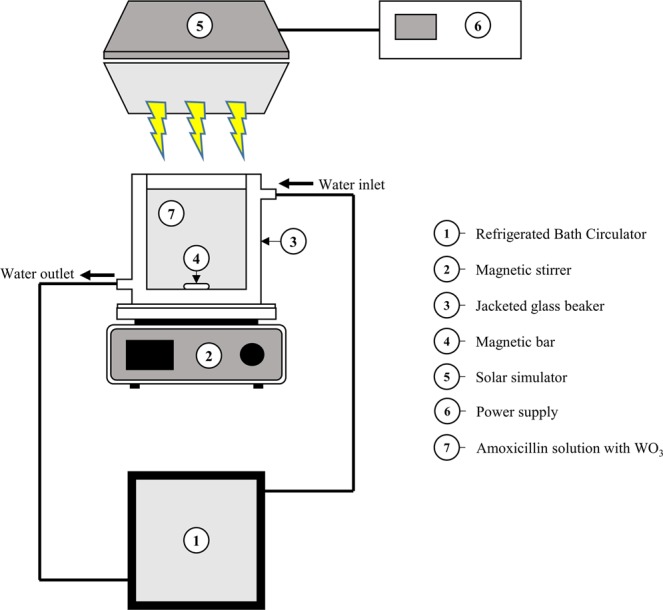
Experimental setup for the solar photocatalytic degradation of AMO.

The photocatalytic solar irradiation set-up consisted of a light source and a photocatalytic reactor (Fig. 2). The light source was a 300-W Xenon lamp installed in a solar simulator (Model: SLB300A, Sciencetech, Canada). The photocatalytic reactor was a 250-mL glass beaker with a double-layer jacket. The solution in the photocatalytic reactor was placed on a magnetic stirrer and uniformly mixed. During the experiments, the temperature was maintained at 25 °C using a refrigerated bath circulator (Daihan Scientific, South Korea). The photocatalytic degradation of AMO was assessed using a 200-mL working solution containing an initial AMO concentration of 1.0, 1.5, or 2.0 μM, a WO_3_ concentration of 0.1, 0.3, or 0.5 g/L, and an initial pH of 4, 6, or 8, which was adjusted using 1 M H_2_SO_4_ and 1 M NaOH. The reaction time was 180 min, and 1-mL aliquots were taken at regular time points (0, 10, 30, 60, 90, 120, 150, and 180 min) for liquid chromatography coupled with mass spectrometry (LC–MS/MS) analysis. All samples were filtered through 0.20-μm PTFE syringe filters to remove WO_3_ and were stored in amber glass vials at 4 °C until analysis. The working solutions were continuously stirred during the experiment.

### Analytical methods

The residual AMO concentration was analyzed using Shimadzu LC-MS 8045. Sample separation was performed using an HSS C18 column (particle size 1.8 μm, 2.1 × 100 mm) with a gradient elution program using a mobile phase consisting of a mixture of water containing 0.1% formic acid and acetonitrile. Table 3 provides details of the analytical conditions. The mineralization of AMO was evaluated by measuring the dissolved organic carbon (DOC) using a TOC-V_CPH_ analyzer (Shimadzu, Japan). The standard solution for DOC calibration was prepared using potassium hydrogen phthalate in the range of 1–20 mgC/L.

**Table 3:** Instrumental conditions for AMO analysis.

Separation conditions			
Instrumentation	Shimadzu LC-MS 8045		
Colum	HSS C18 2.1 × 100 mm I.D.(1.8 µm)		
Mobile phase A	0.1% formic acid in water		
Mobile phase B	Acetonitrile		
Gradient	**Time (min)**	**A**	**B**
	1	90	10
	8	5	95
	12	5	95
	15	90	10
Flow rate	0.2 mL/min		
Injection volume	30 µL		
Ionization mode	ESI		
DL temperature	250 °C		
Capillary temperature	300 °C		
Spary voltage	3000 V		
**Compound**	**Precursor (m/z)**	**Product (m/z)**	**Dwell time (msec)**
Amoxicillin	365.9	349.1	100
	365.9	114	100
	365.9	207.95	100

The photocatalytic degradation kinetics of AMO can be described with a pseudo-first-order kinetic model, as expressed in Eq. (2.1). The degradation and mineralization efficiency in terms of AMO and DOC removal were determined using Eq. (2.2). In addition, the change in degradation efficiency (∆_degradation_) and change in the pseudo-first-order rate constant (∆_k_) of AMO under different experimental conditions were calculated using Eqs (2.3) and (2.4), respectively.

Degradation kinetics

21 $${\rm{l}}{\rm{n}}(\frac{{{\rm{C}}}_{0}}{{\rm{C}}})={\rm{k}}{\rm{t}}$$

AMO or DOC removal efficiency (%)

2.2 $${\rm{AMO}}\,{\rm{Removal}}\,{\rm{or}}\,\mathrm{mineralization}\,( \% )=\frac{{{\rm{C}}}_{0}-{\rm{C}}}{{{\rm{C}}}_{0}}\times 100$$

Degradation efficiency change (%) = ∆_degradation_ (%)

2.3 $${{\rm{\Delta }}}_{{\rm{degradation}}}( \% )={\rm{AMO}}\,{{\rm{removal}}}_{{\rm{i}}}-{\rm{AMO}}\,{{\rm{removal}}}_{{\rm{j}}}$$

Pseudo-first-order rate constant change = ∆_k_

2.4 $${{\rm{\Delta }}}_{{\rm{k}}}=\frac{{{\rm{k}}}_{{\rm{i}}}}{{{\rm{k}}}_{{\rm{j}}}}$$

where *C*_0_ (µМ) is the initial AMO concentration, *C* (µМ) is the AMO concentration at time *t*, *k* (min^−1^) is the pseudo-first-order rate constant, *t* (min) is the reaction time, *AMO removal*_*i*_ and *AMO removal*_*j*_ are the degradation efficiency (%) under the experimental conditions *i* and *j*, respectively, and *k*_*i*_ and *k*_*j*_ are the pseudo-first-order rate constants (min^−1^) under the experimental conditions *i* and *j*, respectively. Mineralization based on DOC was determined after 180 min.

### Experimental design

In this study, RSM based on BBD was employed to optimize the photocatalytic degradation of AMO. The RSM process can be divided into five stages (Fig. S2(a)): (1) selecting independent variables and possible responses, (2) selecting an experimental design strategy, (3) conducting the experiments and recording the results, (4) fitting a model equation to the experimental data and producing response surface graphs, and (5) determining the optimal operating conditions. A three-factor-three-level BBD consisting of 30 experimental runs was implemented in the present study. Three main factors associated with the operating conditions were chosen as independent variables: (A) initial AMO concentration (µМ), (B) catalyst dosage (g/L), and (C) pH. The ranges and levels of the independent variables determined by BBD are presented in Table 4 (Fig. S2(b)).

**Table 4:** Experimental ranges and levels of the independent operating variables.

Original factors	Symbol	Unit	Range	Coded Levels		
				−1	0	1
Initial AMO concentration (C_0_)	A	(μM)	1.0–2.0	1.0	1.5	2.0
Catalytic dosage (WO_3_)	B	(g/L)	0.1–0.5	0.1	0.3	0.5
pH	C	—	4–8	4	6	8

The experimental data were analyzed using Minitab statistical software (Version 18, Minitab Inc., State College, PA) and fitted to a second-order (quadratic) polynomial model as follows:

2.5 $${\rm{Y}}={\beta }_{0}+{\sum }_{{\rm{i}}=1}^{{\rm{k}}}{\beta }_{{\rm{i}}}{{\rm{X}}}_{{\rm{i}}}+{\sum }_{{\rm{i}}=1}^{{\rm{k}}}\,{\sum }_{{\rm{j}}=1}^{{\rm{k}}}{\beta }_{{\rm{i}}{\rm{j}}}\,{{\rm{X}}}_{{\rm{i}}}{{\rm{X}}}_{{\rm{j}}}\,+{\sum }_{{\rm{i}}=1}^{{\rm{k}}}{\beta }_{{\rm{i}}{\rm{i}}}\,{{\rm{X}}}^{2}+{{\rm{e}}}_{0}$$

where *Y* is the predicted response, *X*_*i*_ and *X*_*j*_ are the independent variables, ${\beta }_{0}$ is the constant coefficient, ${\beta }_{i}$ is the linear coefficient, ${\beta }_{ij}\,$is the interaction coefficient, and ${\beta }_{ii}$ is the quadratic coefficient. For statistical calculations, the real values of the variables were transformed into coded values using the following equation^48^:

2.6 $${x}_{i}=\frac{{{\rm{X}}}_{{\rm{i}}}-{{\rm{X}}}_{0}}{\delta {\rm{X}}}$$

where ${X}_{0}$ is the real value of the independent variable at the center point, ${X}_{i}$ is the real value of the independent variable, and $\delta X$ is the step change between the low (−1) and high (+1) levels (as shown in Table 4).

In this study, we have three independent variables (denoted as A, B, and C for initial AMO concentration, catalyst dosage, and pH, respectively), and the mathematical relationship model is written exclusively as

2.7 $${\rm{Y}}={{\rm{b}}}_{0}+{{\rm{b}}}_{1}{\rm{A}}+{{\rm{b}}}_{2}{\rm{B}}+{{\rm{b}}}_{3}{\rm{C}}+{{\rm{b}}}_{12}{\rm{A}}{\rm{B}}+{{\rm{b}}}_{13}{\rm{A}}{\rm{C}}+{{\rm{b}}}_{23}{\rm{B}}{\rm{C}}+{{\rm{b}}}_{11}{{\rm{A}}}^{2}+{{\rm{b}}}_{22}{{\rm{B}}}^{2}+{{\rm{b}}}_{33}{{\rm{C}}}^{2}$$

where *Y* is the predicted response, *b*_0_ is the model constant, *b*_1_, *b*_2_, and *b*_3_ are the linear coefficients, *b*_12_, *b*_13_, and *b*_23_ are the interaction coefficients, and *b*_11_, *b*_22_, and *b*_33_ are the quadratic coefficients.

To evaluate the full quadratic approximation of the BBD response surface model, an analysis of variance (ANOVA) for the experimental response was conducted. The F-values and associated *p*-values were used to determine the order of the model (linear, square, or full quadratic)^51^. A multiple regression analysis was performed to fit the response function to the experimental data. The significance of each coefficient was determined from the *t*-values and associated *p*-values. Coefficients with *t*-values greater than 95% or *p*-values smaller than 0.05 were considered statistically significant.

## Results and Discussion

### Photocatalytic degradation of amoxicillin

#### Photocatalytic degradation kinetics

**Figure 3:**
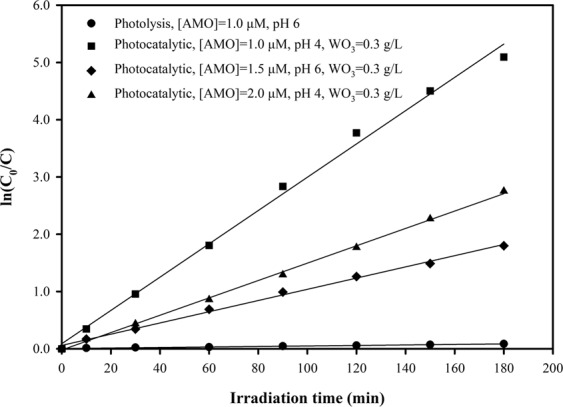
Photolysis and photocatalytic degradation kinetics of AMO.

The photodegradation kinetics of AMO followed a pseudo-first-order kinetic model by plotting ln(C/C_0_) against time (*t*) at different concentrations, as expressed by Eq. (2.1). The pseudo-first-order rate constant (*k*) was determined from the slope of the linear regression line of ln (C_0_/C) versus time. The pseudo-first-order rate constants and the linear regression coefficient (R^2^) for the photodegradation kinetics of AMO are given in Fig. 3 and Table 5.

**Table 5:** Pseudo-first-order rate constants (*k*) and linear regression coefficients (R^2^) for the photolysis and photocatalytic degradation of AMO.

No.	Experimental	Pseudo-first-order rate constant, *k* (min^−1^)	Linear regression coefficient (R^2^)
1	Photolysis, [AMO] = 1.0 μM, pH 6	0.045 × 10^−2^	0.9897
2	Photocatalytic, [AMO] = 1.0 μM, pH 4	2.908 × 10^−2^	0.9955
4	Photocatalytic, [AMO] = 1.5 μM, pH 6	0.979 × 10^−2^	0.9963
5	Photocatalytic, [AMO] = 2.0 μM, pH 4	1.516 × 10^−2^	0.9987

As seen in Fig. 3 and Table 5, degradation plots for the different operating conditions exhibited an almost linear relationship (R^2^ ≥ 0.989). This indicates that the degradation of AMO in both simulated solar photolysis and WO_3_/simulated solar photocatalysis follows pseudo-first-order kinetics with high linear regression coefficients. It was also found that WO_3_ photocatalysis was much more efficient than direct photolysis in terms of AMO degradation as the pseudo-first-order rate constants for WO_3_ photocatalysis increased at least 22-fold compared to that for direct photolysis (Table 5). Furthermore, the photocatalytic degradation of AMO increased with reaction time, with degradation occurring particularly rapidly in the first 90 minutes before slowing. For example, for the experimental conditions of 1.0 µМ of C_0_, 0.3 g/L of WO_3_, and a pH of 4, the AMO degradation rate was rapid at first, reaching 94.14% after the first 90 min of irradiation, before finally reaching 99.39% after 180 min.

#### Effect of initial amoxicillin concentration

**Figure 4:**
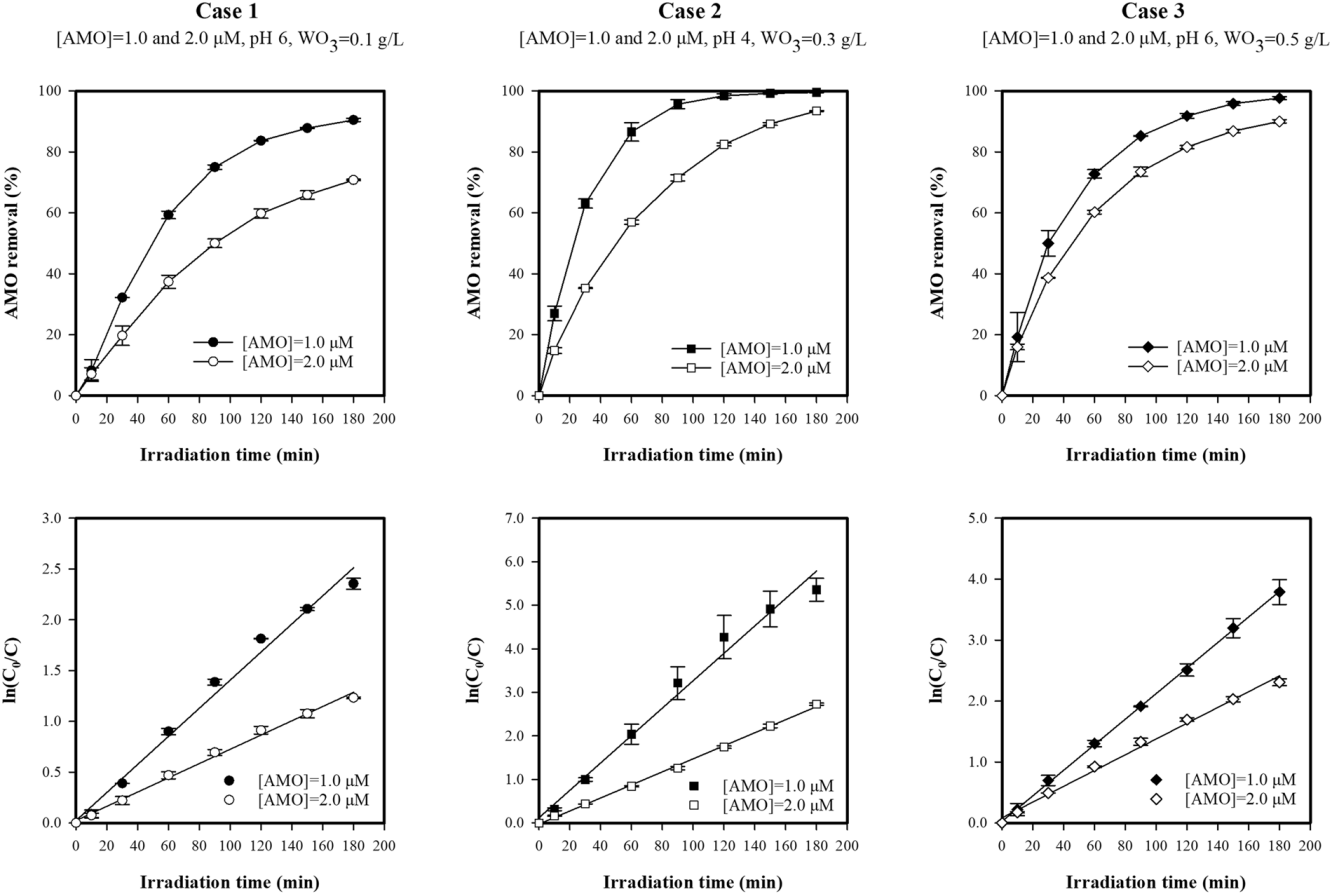
Effect of initial concentration on photocatalytic degradation of AMO.

To investigate the effects of initial AMO concentration (C_0_) on photocatalytic degradation, experiments with initial concentrations of 1.0 and 2.0 µМ were carried out. As shown in Figs 4 and S3, the AMO degradation efficiency decreased with an increase in C_0_ when the other two parameters (catalyst dosage and pH) were unchanged. AMO degradation was found to follow a pseudo-first-order kinetic model in all cases. Pseudo-first-order rate constants (*k*) were calculated from the slope of degradation plots (R^2^ > 0.986), and it was found that they decreased as C_0_ increased. More specifically, as the C_0_ increased from 1.0 µМ to 2.0 µМ, the degradation efficiency and pseudo-first-order rate constants decreased, as calculated using Eqs (2.3) and (2.4):

Case 1 (pH 6, WO_3_ = 0.1 g/L): ∆_degradation_ = −19.69% (from 90.49% at C_0_ = 1.0 µМ to 70.79% at C_0_ = 2.0 µМ) and ∆_k_ = 2.0-fold decrease (from 0.0138 min^−1^ at C_0_ = 1.0 µМ to 0.0070 min^−1^ at C_0_ = 2.0 µМ).

Case 2 (pH 4, WO_3_ = 0.3 g/L): ∆_degradation_ = −6.05% (from 99.51% at C_0_ = 1.0 µМ to 93.46% at C_0_ = 2.0 µМ) and ∆_k_ = 2.1-fold decrease (from 0.0315 min^−1^ at C_0_ = 1.0 µМ to 0.0150 min^−1^ at C_0_ = 2.0 µМ).

Case 3 (pH 6, WO_3_ = 0.5 g/L): ∆_degradation_ = −7.65% (from 97.69% at C_0_ = 1.0 µМ to 90.04% at C_0_ = 2.0 µМ) and ∆_k_ = 1.6-fold decrease (from 0.0210 min^−1^ at C_0_ = 1.0 µМ to 0.0129 min^−1^ at C_0_ = 2.0 µМ).

This reduction in both degradation efficiency and the pseudo-first-order rate constants could be explained by the fact that, when C_0_ increases, the number of available active sites on the WO_3_ surface reduces, thus lowering the generation of ^•^OH and subsequently decreasing the degradation efficiency and corresponding pseudo-first-order rate constant. The reduction in the number of active sites on the WO_3_ surface is the result of three main mechanisms. First, when the C_0_ increases, the AMO molecules absorb more light, reducing the number of photons that reach the catalyst surface^32,52,53^. Second, the absorption of AMO molecules onto the catalyst surface increases with C_0_, thereby reducing the number of available active sites on the catalyst surface. Finally, more intermediates are generated at a higher C_0_, and these intermediates compete with the AMO molecules for active sites on the catalyst surface, which may inhibit the degradation of AMO^52^.

#### Effect of catalyst dosage

**Figure 5:**
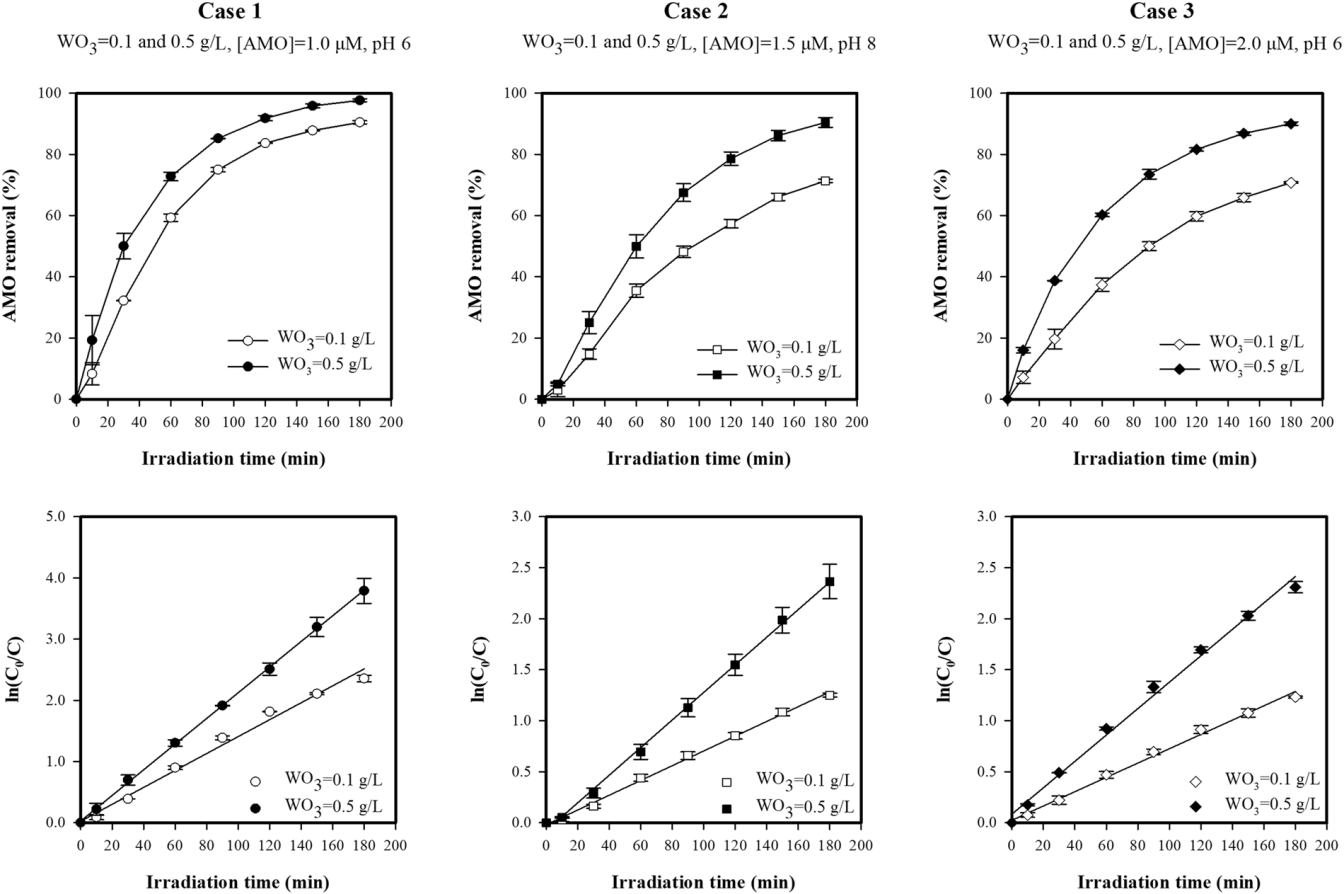
Effect of catalyst dosage on photocatalytic degradation of AMO.

The effect of catalyst dosage on the photocatalytic degradation of AMO was also investigated (Figs 5 and S4), and it was found that the effect of different catalyst dosages was described by a pseudo-first-order kinetic model. The rate constants (*k*) were calculated from the slopes of the resulting linear plots (R^2^ > 0.988). Figure 5 shows that both the AMO degradation efficiency and pseudo-first-order rate constants increased considerably with increasing catalyst dosage. Using Eqs (2.3) and (2.4), the effect of the increase in catalyst dosage from 0.1 g/L to 0.5 g/L on AMO efficiency and *k* are outlined below:

Case 1 (pH 6, C_0_ = 1.0 µМ): ∆_degradation_ = 7.20% (from 90.49% at WO_3_ = 0.1 g/L to 97.69% at WO_3_ = 0.5 g/L) and ∆_k_ = 1.5-fold increase (from 0.0138 min^−1^ at WO_3_ = 0.1 g/L to 0.0210 min^−1^ at WO_3_ = 0.5 g/L).

Case 2 (pH 8, C_0_ = 1.5 µМ): ∆_degradation_ = 19.09% (from 71.36% at WO_3_ = 0.1 g/L to 90.45% at WO_3_ = 0.5 g/L) and ∆_k_ = 1.9-fold increase (from 0.0072 min^−1^ at WO_3_ = 0.1 g/L to 0.0135 min^−1^ at WO_3_ = 0.5 g/L).

Case 3 (pH 6, C_0_ = 2.0 µМ): ∆_degradation_ = 19.25% (from 70.79% at WO_3_ = 0.1 g/L to 90.04% at WO_3_ = 0.5 g/L) and ∆_k_ = 1.8-fold increase (from 0.0070 min^−1^ at WO_3_ = 0.1 g/L to 0.0129 min^−1^ at WO_3_ = 0.5 g/L).

It is clear that the higher the dose of the catalyst, the greater number of active sites on the catalyst surface available, thus facilitating the formation of ^•^OH, and subsequently increasing the removal efficiency and rate constants^31,54^.

#### Effect of pH

**Figure 6:**
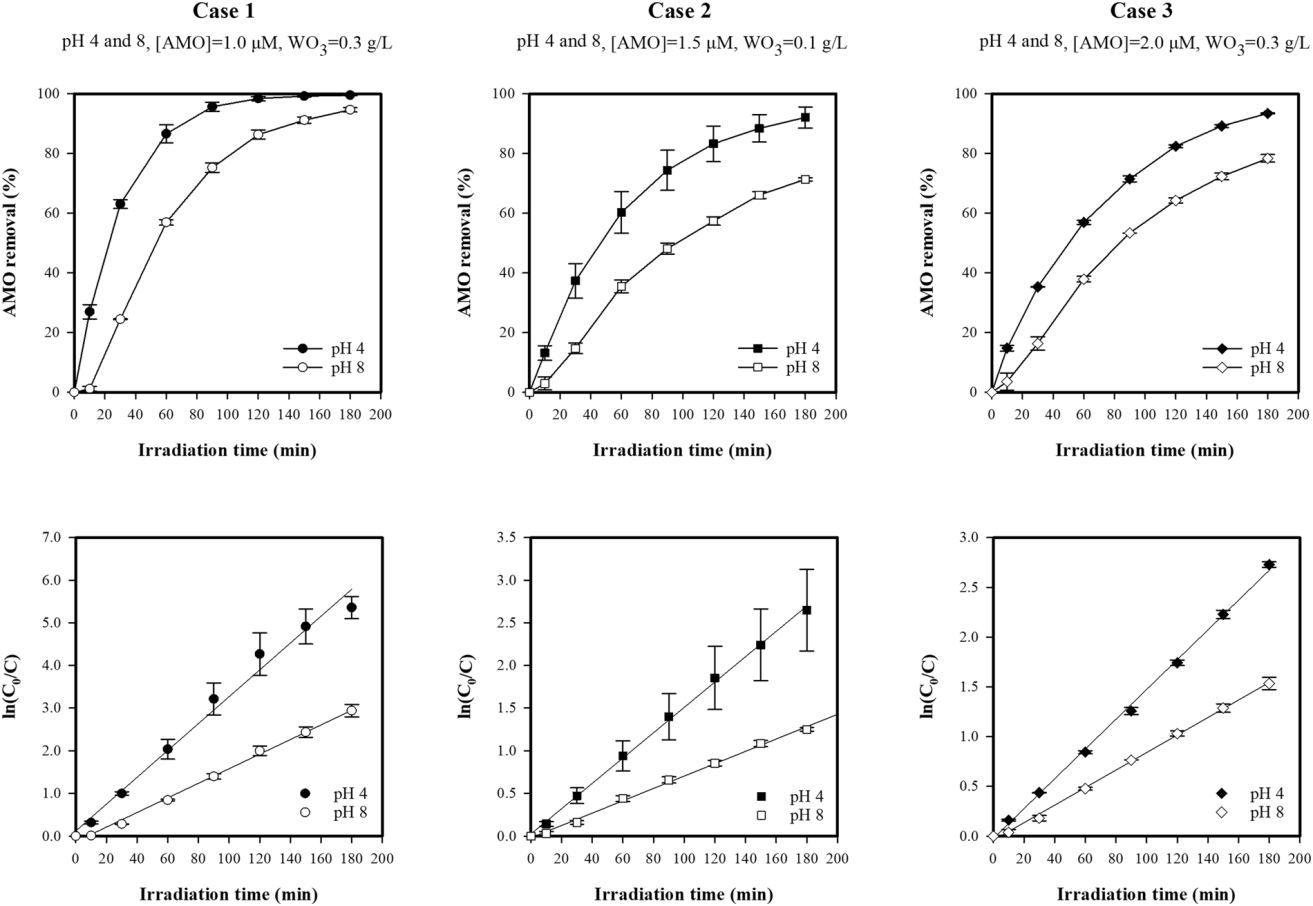
Effect of pH on photocatalytic degradation of AMO.

The pH of the aqueous solution is an important parameter in the photocatalytic degradation of organic compounds because it determines the charge of the catalyst molecules, the size of the aggregates, the charge of the organic pollutants, the adsorption of the organic pollutants onto the catalyst surface, and the concentration of ^•^OH radicals^54,55^. To investigate the effect of pH on the photocatalytic degradation of AMO, experiments with an initial pH of 4 and 8 were conducted (Figs 6 and S5). It was observed that the photocatalytic reaction followed a pseudo-first-order reaction. Figure 6 shows that the removal efficiency and the rate constants decreased with an increase in pH, as calculated using Eqs (2.3) and (2.4):

Case 1 (C_0_ = 1.0 µМ, WO_3_ = 0.3 g/L): ∆_degradation_ = 4.87% (from 99.51% at pH 4 to 94.64% at pH 8) and ∆_k_ = 1.8-fold decrease (from 0.0315 min^−1^ at pH 4 to 0.0171 min^−1^ at pH 8).

Case 2 (C_0_ = 1.5 µМ, WO_3_ = 0.1 g/L): ∆_degradation_ = 20.73% (from 92.09% at pH 4 to 71.36% at pH 8) and ∆_k_ = 2.1-fold decrease (from 0.0149 min^−1^ at pH 4 to 0.0072 min^−1^ at pH 8).

Case 3 (C_0_ = 2.0 µМ, WO_3_ = 0.3 g/L): ∆_degradation_ = 15.08% (from 93.46% at pH 4 to 78.38% at pH 8) and ∆_k_ = 1.7-fold decrease (from 0.0150 min^−1^ at pH 4 to 0.0088 min^−1^ at pH 8).

This can be explained by changes to the surface charges of WO_3_ and AMO. The pH at the point of zero charge (pH_ZPC_) for WO_3_ is 1.9^54^. Therefore, the WO_3_ surface is negatively charged above a pH of 1.9. On the other hand, ionic AMO species are positively charged at acidic pH and negatively charged at alkaline pH^23,56,57^. At an acidic pH, AMO and WO_3_ have opposing charges, thus the electrostatic attraction between the AMO molecules and the catalyst surface increases the absorption rate of AMO onto the WO_3_ surface, leading to an increase in degradation efficiency and the rate constants. At an alkaline pH, both AMO and WO_3_ are negatively charged, leading to repulsive forces between the AMO molecules and the WO_3_ surface. Thus, adsorption onto the surface of WO_3_ is limited, meaning lower degradation efficiency and rate constants. Therefore, when the pH increased from 4 to 8, the repulsive forces hindered the absorption of AMO onto the WO_3_ surface. As a result, the AMO degradation efficiency and pseudo-first-order rate constants decreased.

#### Mineralization of amoxicillin

To assess the photocatalytic degradation of AMO in terms of mineralization, DOC was measured. Of the 30 experimental runs using three-factor-three-level BBD, the highest DOC removal rate was 35.82% at a C_0_ of 1.5 µМ, a WO_3_ dosage of 0.1 g/L, and a pH of 4. Although AMO removal was high under the same conditions (95.61%), DOC removal was quite low, with a ~40% lower removal rate. This was due to the formation of intermediates that could not be completely mineralized under these experimental conditions. Other studies have reported similar discrepancies between AMO and DOC removal using different AOPs (Table 1). Elmolla *et al*.^23^ used UV/H_2_O_2_/TiO_2_ photocatalysis under UVA irradiation and reported the complete degradation of AMO in 20 min and DOC removal of 13.9% after 300 min. Similarly, Elmolla *et al*.^25^ adopted a UV/ZnO photocatalytic process and achieved complete AMO degradation, while DOC removal was only 9.7% after 180 min. Andreozzi *et al*.^20^ utilized ozonation and obtained a removal rate of more than 90% for AMO, but a total organic carbon (TOC) removal rate of only 18.2% after 20 min. Using Fenton’s reagent treatment, Ay *et al*.^21^ reported complete AMO degradation and a TOC removal rate of 37% after 15 min. Therefore, the degradation of AMO does not necessarily lead to the complete mineralization of the products and transformed intermediates of AMO. It should be kept in mind that intermediate products can be toxic in aquatic environments^58,59^, thus the optimization of the process parameters should also target complete mineralization as an end goal.

### Response surface methodology

#### Box-Behnken design

A three-factor-three-level Box-Behnken design (BBD) consisting of 30 experimental runs was adopted to optimize the experimental data. Three operational parameters – initial AMO concentration (µМ; A), catalyst dosage (g/L; B), and pH (C) – were chosen as independent variables for BBD (Table 4). Because photocatalytic performance was assessed in terms of the degradation and mineralization of AMO, the removal of AMO and DOC were employed as the responses for the experimental runs in the present study. Therefore, four predicted responses – Y_1_ (AMO removal after 30 min), Y_2_ (AMO removal after 90 min), Y_3_ (AMO removal after 180 min), and Y_4_ (DOC removal after 180 min) – were expressed as second-order polynomial equations. The complete Box-Behnken design matrix, with the three independent variables (A, B, and C) and the predicted and experimental values of the four responses (Y_1_, Y_2_, Y_3_, and Y_4_), is presented in Table 6. The second-order polynomial equations for the four predicted responses are provided in Table 7 (Eqs 3.1–3.4).

**Table 6:** Three-factors-three-level Box-Behnken design for the photocatalytic degradation of AMO.

Run	Independent variables			Predicted response models (Y) (%)							
	C_0_ (μM)	Catalyst (g/L)	pH	Y_1_ (30-min AMO removal)		Y_2_ (90-min AMO removal)		Y_3_ (180-min AMO removal)		Y_4_ (DOC removal)	
	A	B	C	Experimental	Predicted	Experimental	Predicted	Experimental	Predicted	Experimental	Predicted
1	−1	1	0	54.26	48.46	85.12	84.63	97.22	96.37	2.21	4.48
2	0	0	0	31.20	34.12	63.55	69.06	83.48	86.97	11.50	9.87
3	−1	0	−1	61.57	58.97	94.14	93.89	99.39	100.68	1.45	4.34
4	0	−1	−1	43.16	36.90	81.15	73.28	95.61	90.87	35.82	34.63
5	0	1	1	21.41	25.54	64.60	68.64	88.85	91.67	31.07	31.46
6	0	0	0	29.12	34.12	62.87	69.06	83.45	86.97	11.34	9.87
7	0	0	0	34.08	34.12	69.51	69.06	87.84	86.97	11.61	9.87
8	0	−1	1	13.05	9.10	46.28	45.77	70.77	71.21	13.73	19.66
9	1	0	1	18.58	20.46	53.41	55.21	77.07	77.21	4.71	1.87
10	1	0	−1	35.22	34.28	72.51	72.05	93.64	93.37	3.71	6.44
11	1	1	0	38.62	34.13	75.03	70.58	90.59	89.98	2.33	5.14
12	1	0	−1	35.51	34.28	70.51	72.05	93.28	93.37	3.15	6.44
13	−1	0	−1	64.57	58.97	97.23	93.89	99.64	100.68	2.15	4.34
14	1	−1	0	22.92	21.31	51.44	50.60	70.55	72.11	6.20	5.18
15	0	1	−1	39.37	44.94	75.28	77.11	94.06	94.12	17.87	13.30
16	1	1	0	38.82	34.13	71.99	70.58	89.49	89.98	4.88	5.14
17	0	−1	−1	31.62	36.90	67.66	73.28	88.57	90.87	35.44	34.63
18	−1	0	1	24.37	25.59	73.66	74.74	93.87	94.73	13.58	12.10
19	−1	0	1	24.64	25.59	76.89	74.74	95.42	94.73	16.65	12.10
20	0	0	0	35.51	34.12	71.52	69.06	88.69	86.97	11.72	9.87
21	0	1	−1	39.17	44.94	74.17	77.11	93.89	94.12	17.84	13.30
22	0	1	1	28.69	25.54	70.42	68.64	92.05	91.67	29.85	31.46
23	1	−1	0	16.53	21.31	48.59	50.60	71.04	72.11	8.17	5.18
24	0	0	0	36.34	34.12	72.04	69.06	88.22	86.97	1.50	9.87
25	−1	−1	0	32.15	36.80	74.26	77.92	90.99	90.54	16.42	13.98
26	0	0	0	38.47	34.12	74.84	69.06	90.11	86.97	11.56	9.87
27	1	0	1	14.15	20.46	53.42	55.21	79.68	77.21	4.12	1.87
28	−1	1	0	45.82	48.46	85.32	84.63	98.15	96.37	2.74	4.48
29	0	−1	1	16.50	9.10	50.04	45.77	71.94	71.21	16.50	19.66
30	−1	−1	0	32.27	36.80	75.73	77.92	89.98	90.54	14.62	13.98

**Table 7:** The predicted response models for the Box-Behnken design.

Predicted response (Y)		Unit	Second order polynomial equations	Eqn.
AMO Removal	Y_1_ (30-min AMO removal)	(%)	$34.12-7.46{\rm{A}}+6.12{\rm{B}}-11.80{\rm{C}}+0.29{\rm{AB}}+4.89{\rm{AC}}+2.10{\rm{BC}}+3.38{{\rm{A}}}^{2}-2.32{{\rm{B}}}^{2}-2.67{{\rm{C}}}^{2}$	(3.1)
	Y_2_ (90-min AMO removal)	(%)	$69.06-10.34{\rm{A}}+6.67{\rm{B}}-9.00{\rm{C}}+3.32{\rm{AB}}+0.58{\rm{AC}}+4.76{\rm{BC}}+4.83{{\rm{A}}}^{2}-2.95{{\rm{B}}}^{2}+0.09{{\rm{C}}}^{2}$	(3.2)
	Y_3_ (180-min AMO removal)	(%)	$86.97-6.12{\rm{A}}+5.93{\rm{B}}-5.53{\rm{C}}+3.01{\rm{AB}}-2.55{\rm{AC}}+4.30{\rm{BC}}+2.41{{\rm{A}}}^{2}-2.12{{\rm{B}}}^{2}+2.12{{\rm{C}}}^{2}$	(3.3)
	Y_4_ (180-min DOC removal)	(%)	$9.87-2.03{\rm{A}}-2.38{\rm{B}}+0.80{\rm{C}}+2.37{\rm{AB}}-3.08{\rm{AC}}+8.28{\rm{BC}}-10.62{{\rm{A}}}^{2}+7.95{{\rm{B}}}^{2}+6.94{{\rm{C}}}^{2}$	(3.4)

A, B, and C are the independent variables (in term of coded) of the initial AMO concentration (μM), catalytic dosage (g/L), and pH, respectively.

The quality of the model fit was evaluated using analysis of variance (ANOVA); the results are presented in Table 8. The ANOVA revealed that the four second-order quadratic regression models were highly significant, because the with F-values (17.52, 28.40, 48.02, and 21.14 for the Y_1_, Y_2_, Y_3_, and Y_4_ models, respectively) greater than the tabular F-value for α = 0.05 (F_0.05(9,9) tabular_ = 3.18^60^). The *p*-values of the four models were very low (0.0000 for all models), which confirms that the models were statistically significant at a 5% level. The F-value is a statistically valid measure of how well the factors describe the variation in the data in that the mean and estimated factor effects are real. The *p*-value is relatively low (*p* < 0.05), demonstrating the significance of the model. Thus, with their large F-values and small *p*-values, the four models explain the measured data well, with the corresponding coefficients demonstrating high significance.

**Table 8:** ANOVA results from the response surface quadratic models for AMO removal (%) and DOC removal (%).

Source	Sum of squares	Degree of freedom	Mean square	F-value	*p*-value
**Y**_**1**_ **, 30-min AMO removal**					
Model	4134.33	9	459.37	17.52	0.000
Residual	524.45	20	26.22		
Lack of fit	295.88	3	98.63	7.34	0.002
Pure error	228.58	17	13.45		
Total	4658.78	29			
R_squared_ = 0.8874, Adjusted R_squared_ = 0.8368, Predicted R_squared_ = 0.7261					
**Y**_**2**_ **, 90-min AMO removal**					
Model	4243.92	9	471.55	28.40	0.000
Residual	332.02	20	16.6		
Lack of fit	77.5	3	25.83	1.73	0.200
Pure error	254.52	17	14.97		
Total	4575.94	29			
R_squared_ = 0.9274, Adjusted R_squared_ = 0.8948, Predicted R_squared_ = 0.8430					
**Y**_**3**_ **, 180-min AMO removal**					
Model	2055.42	9	228.38	48.02	0.000
Residual	95.11	20	4.756		0.288
Lack of fit	18.44	3	6.146	1.36	
Pure error	76.68	17	4.51		
Total	2150.53	29			
R_squared_ = 0.9558, Adjusted R_squared_ = 0.9359, Predicted R_squared_ = 0.9075					
**Y**_**4**_ **, 180-min DOC removal**					
Model	2621.21	9	291.246	21.14	0.000
Residual	275.49	20	13.775		
Lack of fit	174.44	3	58.147	9.78	0.001
Pure error	101.05	17	5.944		
Total	2896.71	29			
R_squared_ = 0.9049, Adjusted R_squared_ = 0.8621, Predicted R_squared_ = 0.7891					

To evaluate whether the models could successfully be used for prediction, the lack of fit (LOF) was assessed. The LOF for the models Y_1_ and Y_4_ was statistically significant (*p* < 0.05), meaning there was a greater than 5% chance of prediction failure compared to experimental data for AMO removal after 30 min and DOC removal after 180 min. In contrast, the LOF for the models Y_2_ and Y_3_ was statistically insignificant (*p* = 0.200 and *p* = 0.288, respectively), meaning Y_2_ and Y_3_ can be successfully used for prediction and optimization. Furthermore, to determine whether the models fit the experimental data well and whether the independent variables had a significant effect on the responses, coefficients of determination (R^2^) were used. R^2^ represents the proportion of the variation in the response that is explained by a model and is a statistical measure used for the goodness-of-fit for a model^61^. R^2^ always falls between 0 and 1. The closer R^2^ is to 1, the more closely the model fits the experimental data and the better it can predict the response^61,62^. In the present study, R^2^ was found to be 0.8874, 0.9274, 0.9558, and 0.9049 for the Y_1_, Y_2_, Y_3_, and Y_4_ models, respectively, indicating that the quadratic polynomial models were a good fit for the experimental results.

Adjusted and predicted R^2^ also explain the goodness-of-fit for a model. Adjusted R^2^ is used to compare the goodness-of-fit for a regression model that contains a differing number of intendant variables. A high value for adjusted R^2^ indicates high significance, and the smaller the gap between R^2^ and adjusted R^2^, the stronger the goodness-of-fit for a model^61^. Predicted R^2^ is used to determine how well a regression model is able to make a response prediction. In the present study, adjusted R^2^ was 0.8368, 0.8948, 0.9359, and 0.8621 for the Y_1_, Y_2_, Y_3_, and Y_4_ models, representing high significance. Likewise, the small gap between R^2^ and predicted R^2^ shown in Table 8 indicates that the models almost perfectly explained the variation in the experimental data and can thus be used for prediction.

**Figure 7:**
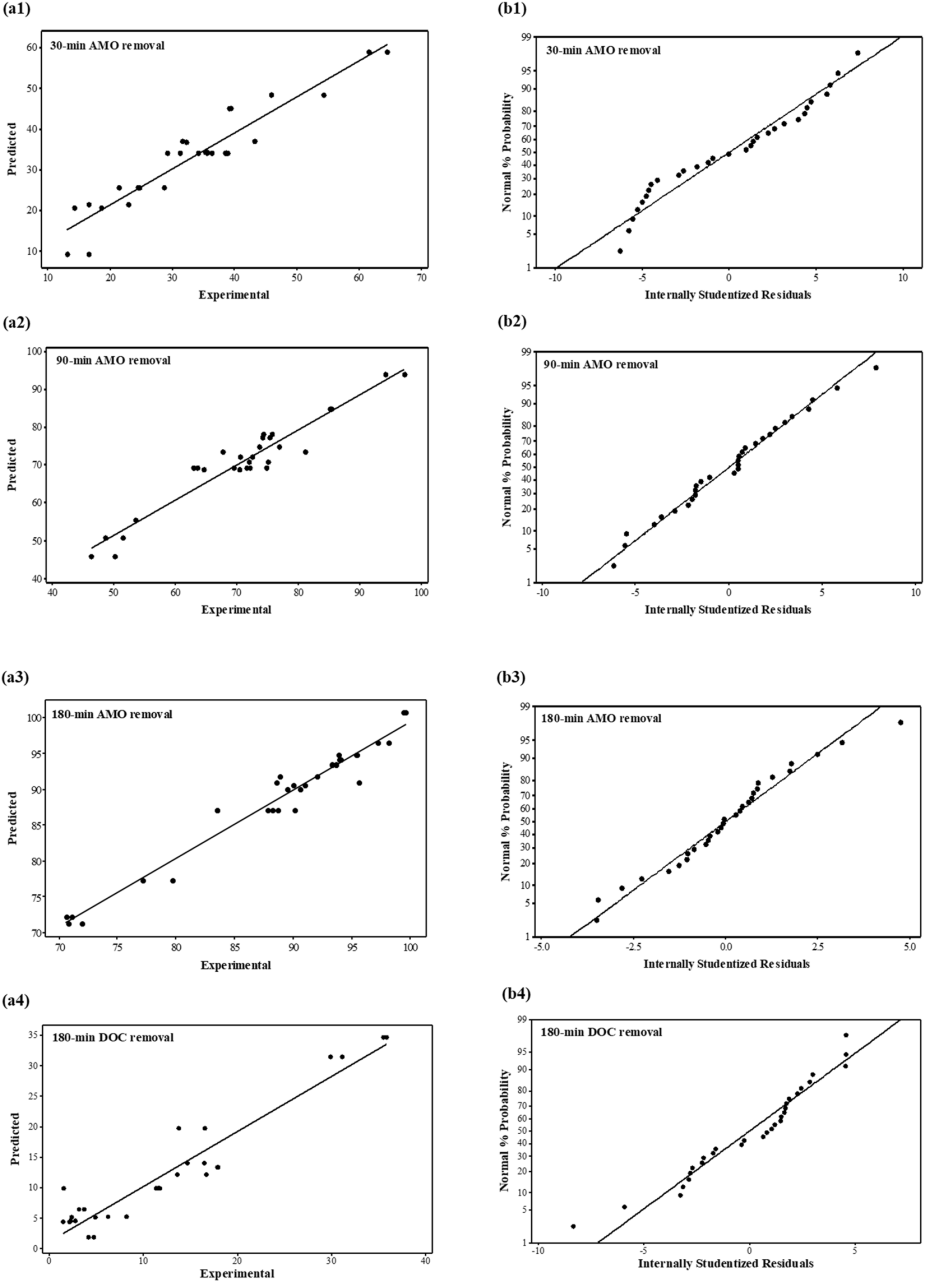
Diagnostic plots for the photocatalytic degradation of AMO: (**a**) Experimental and predicted values for BBD; (**b**) the normal % probability and internally studentized residuals.

In addition, the diagnostic plots presented in Fig. 7, which were employed to determine the residual analysis of the response surface design, confirmed the close association between the statistical assumptions and the analyzed data. The experimental data and the responses predicted by the models are compared in Fig. 7(a). Plots of normal probability versus internally studentized residuals, an important diagnostic tool to determine whether the assumptions underlying the statistical analysis are met, were constructed for the four models (Fig. 7(b)). As seen in Fig. 7, the predicted and experimental results were in good agreement. Therefore, the ANOVA, LOF, and R^2^ results together confirmed that all of four models were statistically significant and could be used to predict the degradation of AMO using photocatalysis.

The quantitative effects of the independent variables in each of the four models were evaluated using the ANOVA results and percentage contributions (PCs) for each individual factor (Table 9). The PCs for each individual variable were calculated using the sum of squares (SS), and the total percentage contributions (TPCs) for the first-order, interaction, and quadratic terms were determined using Eqs (3.5–3.7) ^61,63^

3.5 $${{\rm{TPC}}}_{{\rm{i}}}=\frac{{\sum }_{{\rm{i}}=1}^{{\rm{n}}}\,{{\rm{SS}}}_{{\rm{i}}}}{{\sum }_{{\rm{i}}=1}^{{\rm{n}}}\,{\sum }_{{\rm{j}}=1}^{{\rm{n}}}\,{{\rm{SS}}}_{{\rm{i}}}+{{\rm{SS}}}_{{\rm{ii}}}+{{\rm{SS}}}_{{\rm{ij}}}}\times 100$$

3.6 $${{\rm{TPC}}}_{{\rm{ij}}}=\frac{{\sum }_{{\rm{i}}=1}^{{\rm{n}}}{\,\sum }_{{\rm{j}}=1}^{{\rm{n}}}\,{{\rm{SS}}}_{{\rm{ij}}}}{{\sum }_{{\rm{i}}=1}^{{\rm{n}}}{\,\sum }_{{\rm{j}}=1}^{{\rm{n}}}\,{{\rm{SS}}}_{{\rm{i}}}+{{\rm{SS}}}_{{\rm{ii}}}+{{\rm{SS}}}_{{\rm{ij}}}}\times 100$$

3.7 $${{\rm{TPC}}}_{{\rm{ii}}}=\frac{{\sum }_{{\rm{i}}=1}^{{\rm{n}}}{\,\sum }_{{\rm{i}}=1}^{{\rm{n}}}\,{{\rm{SS}}}_{{\rm{ii}}}}{{\sum }_{{\rm{i}}=1}^{{\rm{n}}}{\,\sum }_{{\rm{j}}=1}^{{\rm{n}}}\,{{\rm{SS}}}_{{\rm{i}}}+{{\rm{SS}}}_{{\rm{ii}}}+{{\rm{SS}}}_{{\rm{ij}}}}\times 100$$

where *TPC*_*i*_, *TPC*_*ij*_, and *TPC*_*ii*_ are the total percentage contribution of the first-order, interaction, and quadratic terms, respectively, and *SS*_*i*_, *SS*_*ij*_, and *SS*_*ii*_ are the computed sum of squares for the first-order, interaction, and quadratic terms, respectively.

**Table 9:** ANOVA results for the four quadratic models for AMO photocatalytic degradation.

Quadratic model	Factor	Coefficient	F-value	*p*-value	Sum of squares	Percentage contribution (%)
**Y**_**1**_ (30-min AMO removal)	Intercept	34.12				
	A	−7.46	33.92	0.0000	889.35	21.58
	B	6.12	22.87	0.0000	599.60	14.55
	C	−11.80	84.95	0.0000	2227.52	54.05
	A^2^	3.38	3.22	0.0880	84.35	2.05
	B^2^	−2.32	1.52	0.2320	39.90	0.97
	C^2^	−2.67	2.01	0.1710	52.80	1.28
	AB	0.29	0.03	0.8730	0.68	0.02
	AC	4.89	7.30	0.0140	191.48	4.65
	BC	2.10	1.35	0.2600	35.28	0.86
**Y**_**2**_ (90-min AMO removal)	Intercept	69.06				
	A	−10.34	103.05	0.0000	1710.79	40.48
	B	6.67	42.94	0.0000	712.79	16.87
	C	−9.00	77.99	0.0000	1294.69	30.63
	A^2^	4.83	10.36	0.0040	171.97	4.07
	B^2^	−2.95	3.86	0.0630	64.14	1.52
	C^2^	0.09	0.00	0.9520	0.06	0.00
	AB	3.32	5.30	0.0320	87.96	2.08
	AC	0.58	0.16	0.6920	2.68	0.06
	BC	4.76	10.92	0.0040	181.22	4.29
**Y**_**3**_ (180-min AMO removal)	Intercept	86.97				
	A	−6.21	129.64	0.0000	616.52	30.08
	B	5.93	118.19	0.0000	562.08	27.43
	C	−5.53	102.74	0.0000	488.62	23.84
	A^2^	2.41	9.00	0.0070	42.82	2.09
	B^2^	−2.12	6.99	0.0160	33.27	1.62
	C^2^	2.12	7.00	0.0150	33.31	1.63
	AB	3.01	15.25	0.0010	72.53	3.54
	AC	−2.55	10.97	0.0030	52.19	2.55
	BC	4.30	31.13	0.0000	148.06	7.22
**Y**_**4**_ (180-min DOC removal)	Intercept	9.87				
	A	−2.03	4.81	0.0400	66.23	2.66
	B	−2.38	6.59	0.0180	90.8	3.64
	C	0.80	0.74	0.4000	10.18	0.41
	A^2^	−10.62	60.51	0.0000	833.5	33.43
	B^2^	7.95	33.89	0.0000	466.78	18.72
	C^2^	6.94	25.86	0.0000	356.17	14.29
	AB	2.37	3.25	0.0870	44.77	1.80
	AC	−3.08	5.52	0.0290	76.00	3.05
	BC	8.28	39.82	0.0000	548.47	22.00

**Figure 8:**
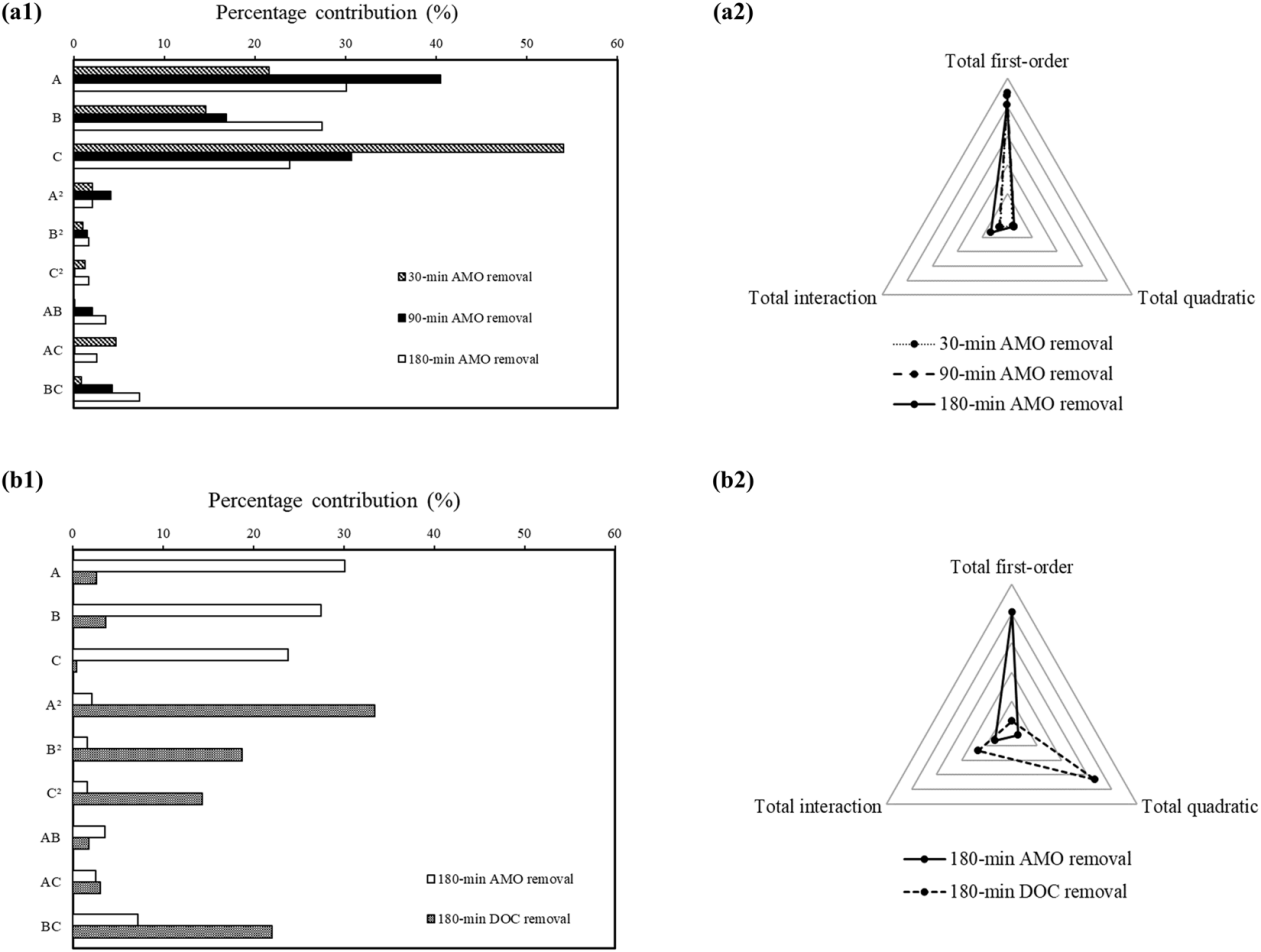
Percentage contributions to AMO photocatalytic degradation: (**a1** and **b1**) Components; (**a2** and **b2**) collective effects of each term.

Figure 8 presents the quantitative effects of the independent variables in the four models on AMO removal (after 30, 90, and 180 min) and DOC removal after 180 min. As seen in Fig. 8(a) and Table 9, as the reaction time increased from 30 min to 180 min, the contributions of initial AMO concentration (A) and catalyst dosage (B) to AMO removal increased by 8.50% and 12.88%, while the contribution of pH (C) decreased by 30.21%. The first-order terms accounted for a higher contribution than the other terms, with a total percentage contribution (TPC_i_) of 90.18% for Y_1_, 87.98% for Y_2_, and 81.35% for Y_3_. The second highest contribution was exhibited by the interaction terms, with a total percentage contribution (TPC_ij_) of 5.52% for Y_1_, 6.43% for Y_2_, and 13.31% for Y_3_. As the reaction time increased from 30 min to 180 min, the contributions of the first-order terms decreased by 8.83%, while those of the quadratic and interaction terms increased by 1.04% and 7.79%, respectively, indicating that the combined effects of the variables on AMO removal increased with increased reaction time, though they were still much less influential than the effects of the variables individually.

In the Y_1_ model (AMO removal after 30 min), the three first-order terms (A, B, and C) and one interaction term (AC) were found to be statistically significant (*p* < 0.05). In particular, pH (C) exhibited the highest level of significance, with a contribution of 54.05%. The TPC_i_ for the first-order terms was 90.18%, with the lowest contribution shown by the quadratics terms.

In the Y_2_ model (AMO removal after 90 min), three first-order terms (A, B, and C) and two interaction terms (AB and BC) were statistically significant (*p* < 0.05). The first-order terms had the highest level of significance (with a contribution of 87.98%), followed by the interaction terms and the quadratics terms (Fig. 8a).

In the Y_3_ model (AMO removal after 180 min), all of the terms (A, B, C, A^2^, B^2^, C^2^, AB, AC, and BC) were statistically significant for AMO removal (*p* < 0.05). Based on the results of the three time-specific models (Y_1_, Y_2_, and Y_3_), it was found that the first-order terms made the greatest contribution to AMO degradation over the entire reaction time. For the three first-order terms, in the early stages of the reaction process (i.e., 30 min), AMO removal was significantly affected by initial pH, with a contribution of 54.05%, while the roles of other two factors became more important as the reaction time increased.

In the 180-min DOC removal model (Y_4_), all of the terms except C (*p* = 0.400) and AB (*p* = 0.087) were statistically significant. In contrast to Y_1_–Y_3_, the first-order terms accounted for only 18.65% of the TPC, while the interaction and quadratic terms exhibited a combined contribution of 93.29% (66.45% and 26.85% for TPC_ii_ and TPC_ij_, respectively; Fig. 8b). This difference from the other models might be because of the unknown concentration of intermediates produced during the reaction. As mentioned above, intermediates containing organic carbon that are present due to the incomplete mineralization of AMO should be taken into consideration by DOC removal models to allow for more accurate prediction. Given the presence of these intermediates, the interaction and quadratic terms became more important to the prediction of DOC removal. More specifically, the quadratic term A^2^ demonstrated the highest contribution (33.43%), followed by B^2^ and C^2^. In addition, BC accounted for 22.00% of the TPC_ij_ of 26.85%.

#### Response surface analysis

Over the 30 experimental runs, the highest AMO degradation was 99.64% with C_0_ = 1.0 µМ, WO_3_ = 0.3 g/L, and pH 4 for a reaction time of 180 min. As a result, the Y_3_ model (with a reaction time of 180 min) was selected and analyzed to determine the optimal conditions based on RSM and BBD for AMO degradation efficiency in the proposed photocatalysis process.

**Figure 9:**
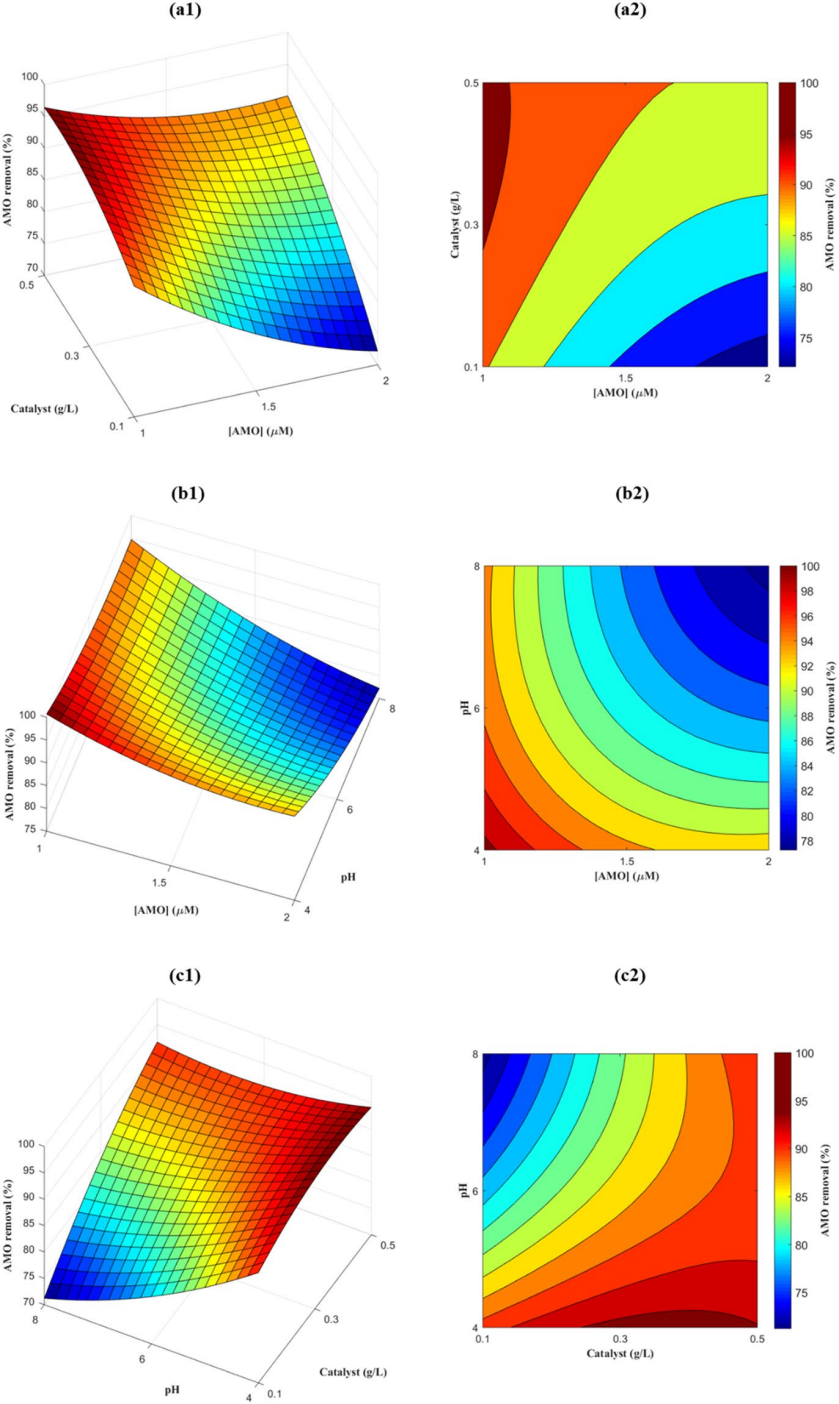
3D response surface graphs and 2D contour plots for AMO photocatalytic degradation.

Three-dimensional (3D) response surface plots and two-dimensional (2D) contour plots for AMO degradation efficiency (%) are presented in Fig. 9 for the following combinations: C_0_ versus catalyst dosage (Fig. 9(a)), C_0_ versus pH (Fig. 9(b)), and catalyst dosage versus pH (Fig. 9(c)).

Figure 9(a) presents the combined effects of initial AMO concentration (A) and catalyst dosage (B) on AMO removal (%). As seen, at a constant catalyst dosage, AMO removal decreased as the initial AMO concentration increased, and this trend became more obvious at lower catalyst dosages. For example, with a catalytic load of 0.1 g/L, AMO removal decreased by 18.42% when AMO C_0_ increased from 1.0 μM to 2.0 μM, while AMO removal only dropped by 6.38% at a dosage of 0.5 g/L. The negative coefficient (−6.21) for A in the response function (Eq. 3.3) also confirmed the antagonistic effect of initial AMO concentration on AMO removal. As mentioned in the previous section, one reason for this inverse relationship between initial AMO concentration and AMO degradation efficiency might be the reduction in the number of available active sites on the photocatalyst surface and the deactivation of the catalyst due to the accumulation of products and reactants on its surface. Conversely, when the catalyst dosage increased, AMO removal increased when the initial AMO concentration was constant. Specifically, at an initial AMO concentration of 1.0 μM, AMO removal increased by 5.84% when catalyst dosage increased from 0.1 g/L to 0.5 g/L. Similarly, at an initial AMO concentration of 2.0 μM, the AMO removal increased by 17.89% for the same increase in catalytic dosage. The positive coefficient (5.93) for B in Eq. (3.3) also confirmed that catalytic dosage had no antagonistic effect on the response. A possible explanation for this may be that there was an increase in the number of available active sites on the photocatalyst, resulting in an increased in ^•^OH generation, and thus greater AMO degradation.

Figure 9(b) summarizes the influence of initial AMO concentration (A) and pH (C) on AMO removal (%) at a constant catalyst dosage (WO_3_ = 0.3 g/L). It can be observed that both initial AMO concentration and pH had a negative effect on AMO removal. At pH 4, AMO removal decreased by 7.29%, compared to a decrease of 17.51% at pH 8, when the initial AMO concentration increased from 1.0 μM to 2.0 μM. Similarly, when pH increased from 4 to 8, AMO removal decreased by 5.94% at an initial AMO concentration of 1.0 μM and by 16.15% at an initial AMO concentration of 2.0 μM. The inverse relationship between these two factors and AMO degradation efficiency was also illustrated by the negative coefficients for A (−6.21) and C (−5.53) in Eq. (3.3).

The interaction effects of catalyst dosage and pH on AMO degradation efficiency are presented in Fig. 9(c). The results show that, at a constant AMO concentration of 1.5 μM, catalyst dosage had a positive effect on AMO removal, while pH had a negative effect. When the pH was increased from 4 to 8, AMO removal decreased by 19.65% and 2.44% at catalyst dosage of 0.1 g/L and 0.5 g/L, respectively. The inverse relationship between pH (C) and AMO degradation was also illustrated by the negative coefficient for C (−5.53) in the response function (Eq. 3.3). In contrast, when the catalyst dosage increased from 0.1 g/L to 0.5 g/L, AMO removal increased by 3.26% at a pH of 4 and by 20.47% at a pH of 8. AMO degradation was directly proportional to catalyst dosage (B), as indicated by the positive coefficient (5.93) for B in Eq. (3.3).

### Optimization of AMO degradation efficiency

One of the main objectives of this study was to find the optimal conditions for the target parameters in order to maximize AMO degradation. Thus, the desired goal was defined as a maximum AMO degradation of 100%. Using Minitab software, the optimal conditions for maximum AMO degradation were found to be an initial AMO concentration of 1.0 μM, a catalyst dosage of 0.104 g/L, and a pH of 4. Under these conditions, AMO and DOC removal after 180 min were predicted to be 99.99% and 24.75%, respectively.

## Conclusions

In this study, the WO_3_-assisted photocatalytic degradation of AMO with three influencing factors (initial AMO concentration, WO_3_ dosage, and pH) was investigated using simulated solar irradiation. Based on AMO and DOC removals, the following conclusions were drawn:

- The photocatalytic degradation of AMO followed pseudo-first-order kinetics. The pseudo-first-order rate constants and degradation efficiencies decreased with increasing initial AMO concentration and pHs, while showed increasing with more WO_3_ dosage. Compared to almost compete AMO degradations, much lower mineralization (i.e., low DOC removals) was found, presumably due to the intermediate products formed via AMO oxidation.
- The second-order polynomial regression models revealed good fit to the experimental data. The total percentage contributions showed that the highest contributions to AMO removals were by the first-order terms, however, in the regression models of DOC removals, contributions by the quadratic terms were significantly increased.
- Initial AMO concentration of 1.0 μM, WO_3_ dosage of 0.104 g/L, and pH of 4 were found to be optimum conditions for complete removal of AMO.

## Supplementary information

Supplementary Information
