# Supplementary material for: Tungsten Trioxide (WO3)-assisted Photocatalytic Degradation of Amoxicillin by Simulated Solar Irradiation
Source: Sci Rep. 2019 Jun 27;9:9349. doi: 10.1038/s41598-019-45644-8 (PMC6597549; doi:10.1038/s41598-019-45644-8)
Supplement: Supplementary file 1 — Supplementary Information [file 41598_2019_45644_MOESM1_ESM.docx]

**Supplementary information**

**Tungsten Trioxide (WO_3_)-assisted Photocatalytic Degradation of Amoxicillin by Simulated Solar Irradiation**

Thao Thi Nguyen, Seong-Nam Nam*, Jooyoung Son, Jeill Oh

Department of Civil and Environmental Engineering, Chung-Ang University, 84, Heukseok-ro, Dongjak-gu, Seoul, 06974, Republic of Korea

*Corresponding authors

Tel.: +82-2-826-4528 (S.-N. Nam)

E-mail: [namsn76@gmail.com](mailto:namsn76@gmail.com) (S.-N. Nam)

**Word count: 8,697**

**Number of Figures: 9**

**Number of Tables: 9**


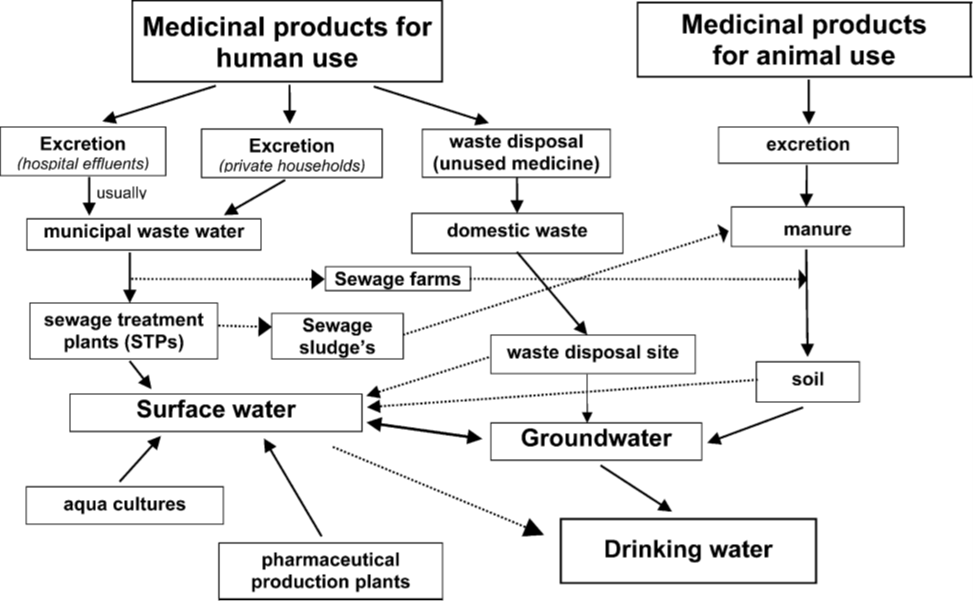


**Figure S1**. Scheme showing possible sources and pathways for the occurrence of pharmaceutical residues in the aquatic environment (Reference 4 in the main manuscript)


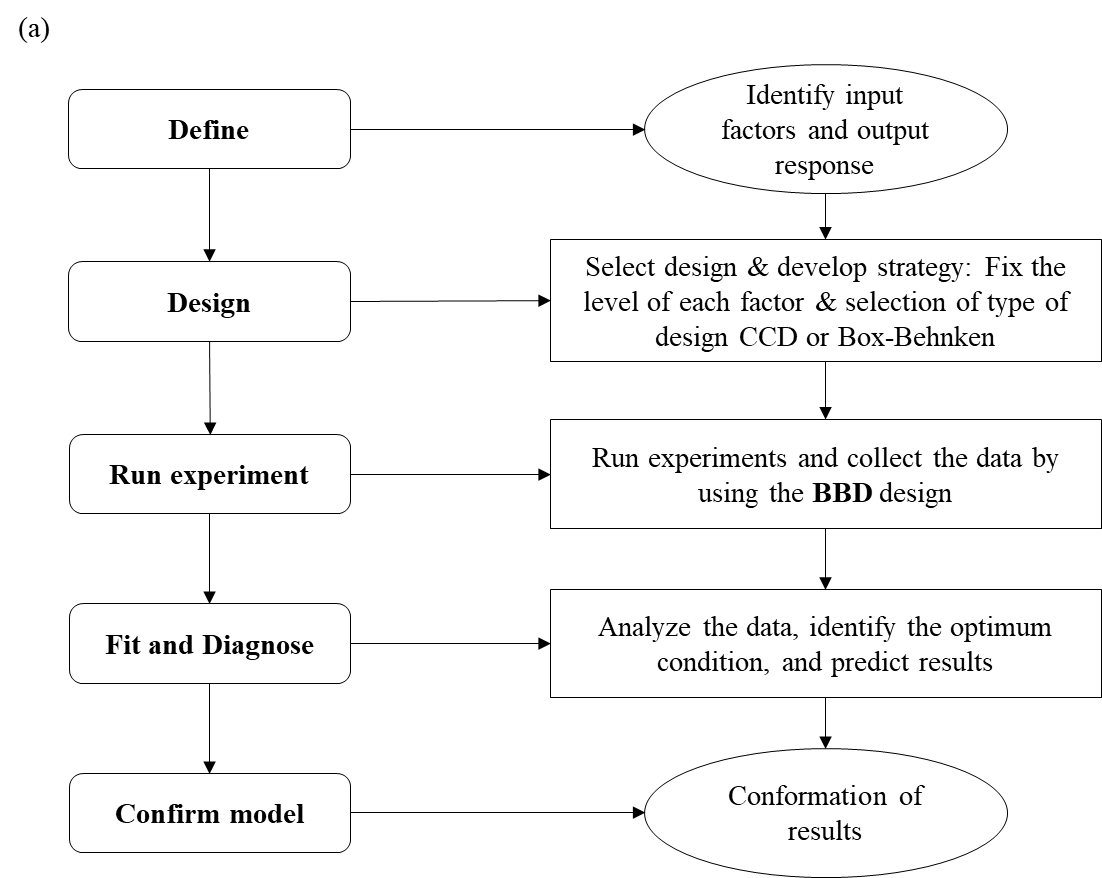


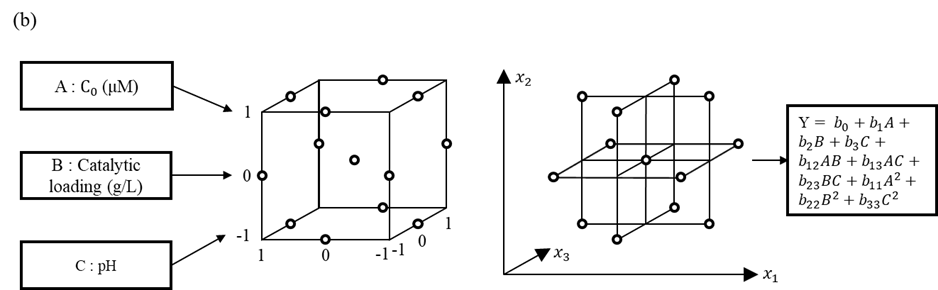


**Figure S2**. AMO photocatalytic degradation experiment design using RSM based on BBD. (a) Design experiment in RSM methodology

**Figure S3**. Effect of initial concentration on photocatalytic degradation of AMO

**Figure S4**. Effect of catalyst dosage on photocatalytic degradation of AMO

**Figure S5**. Effect of pH on photocatalytic degradation of AMO
